# Intraluminal unilateral ectopic ureter associated to ectopic ureterocele in a female dog - clinical, diagnostic and surgical aspects

**DOI:** 10.29374/2527-2179.bjvm008424

**Published:** 2025-03-18

**Authors:** Daniel de Pinho Alves, Maria Eduarda dos Santos Lopes Fernandes, Cecília Azevedo Dias Lopes, Thainá de Lima Risso, Mayara do Nascimento Trindade, Thais Marques Moreira, Isabela Scalioni Gijsen, Rodrigo Pereira da Costa Duarte

**Affiliations:** 1 Departamento de Medicina e Cirurgia Veterinária, Instituto de Veterinária, Universidade Federal Rural do Rio de Janeiro, Seropédica, RJ, Brazil.

**Keywords:** ectopia, dogs, urography, abdominal ultrasound, neoureterostomy, ectopia, cães, urografia, ultrassonografia abdominal, neoureterostomia

## Abstract

Ureteral ectopia is a congenital malformation characterized by the abnormal location of the distal aspect of one or both ureters, being classified according to its anatomical path as intramural or extramural. The most common clinical sign is urinary incontinence. The presence of other associated urogenital anomalies, such as hydroureter, hydronephrosis and ureterocele are possible, being the last one a rare condition characterized as a cystic dilation of the submucosal layer of the distal ureter. The diagnosis is based on patient history, clinical signs and imaging exams. Treatment consists in surgical correction, with the technique variating according to the condition classification, and the prognosis is favorable, however most animals remain incontinent. This paper objective to report the diagnostic and clinical surgical conduction of intramural unilateral ectopic ureter correction associated to ectopic ureterocele in a Siberian Husky, 7-months-old, attended at Veterinary Hospital of Federal Rural University of Rio de Janeiro with complaint of urinary incontinence and clinical history of bacterial cystitis. The diagnosis of intramural unilateral ectopia associated with ureterocele was obtained through abdominal ultrasound and excretory urography, being confirmed with surgery. The surgical technique performed was neoureterostomy, and there were no trans or post-surgical intercurrence. Despite maintenance of the ureterocele and right ureter and renal pelvis dilation two days after surgery, observed during abdominal ultrasound, these alterations has positive evolution one week after the surgical procedure. Patient presented significant improvement of urinary incontinence two months after surgery.

## Resumo

A ectopia ureteral é uma malformação congênita caracterizada pela localização anormal do aspecto distal de um ou ambos os ureteres, sendo classificada de acordo com seu trajeto anatômico como intramural ou extramural. O sinal clínico mais comum é a incontinência urinária. É possível a presença de outras anomalias urogenitais associadas, como hidroureter, hidronefrose e ureterocele, sendo esta última uma condição rara caracterizada como uma dilatação cística da camada submucosa do ureter distal. O diagnóstico é baseado na história do paciente, sinais clínicos e exames de imagem. O tratamento consiste na correção cirúrgica, com a técnica variando de acordo com a classificação da condição, e o prognóstico é favorável, porém a maioria dos animais permanece incontinente. O objetivo deste trabalho foi relatar o diagnóstico e a conduta cirúrgica clínica da correção de ureter ectópico unilateral intramural associado à ureterocele ectópica em um Husky Siberiano, de 7 meses de idade, atendido no Hospital Veterinário da Universidade Federal Rural do Rio de Janeiro com queixa de incontinência urinária e história clínica de cistite bacteriana. O diagnóstico de ectopia unilateral intramural associado à ureterocele foi obtido por meio de ultrassonografia abdominal e urografia excretora, sendo confirmado com cirurgia. A técnica cirúrgica realizada foi a neoureterostomia, não havendo intercorrências trans ou pós-cirúrgicas. Apesar da manutenção da ureterocele e da dilatação do ureter e da pelve renal direita dois dias após a cirurgia, observadas durante ultrassonografia abdominal, essas alterações tiveram evolução positiva uma semana após o procedimento cirúrgico. O paciente apresentou melhora significativa da incontinência urinária dois meses após a cirurgia.

## Introduction

Ureteral ectopia is a congenital abnormality characterized by the abnormal location, outside the vesical trigone region, of the pelvic part of one or both ureters. Intramural ectopic ureters inserted in the vesical trigone but follow an abnormal path inside the bladder wall opening in abnormal regions. This anomaly is more common in young female dogs and has already been described in several breeds (Acierno & Labato, 2019).

Ureterocele is a congenital condition, rare and from unknown origin in dogs, characterized by ureter submucosal layer dilation (Davidson & Westropp, 2014). Ureterocele can be classified as orthotopic or ectopic. Orthotopic ureteroceles are completely contained inside the bladder and have an orifice that communicates with the organ. The ectopics are found in the bladder neck or the urethra and are associated with the presence of ectopic ureter (Rademacher, 2019).

The most common clinical sign associated with ectopic ureter is the intermittent urinary incontinence in puppies. Hematuria, hydronephrosis, ureteral dilation, recurrent cystitis and perivulvar dermatitis are also reported (Fossum, 2021). Ureterocele usually do not show clinical signs, but urinary incontinence and pollakiuria can be observed (Tattersall & Welsh, 2006).

The diagnosis is based on urinary incontinence history associated, very often, to recurrent urinary infection in young animals. In the physical exam it is possible to observe uremic odor, dermatitis, and humidity in the hair of the vulvar or preputial region (McLoughlin & Chew, 2000). Imagining exams as abdominal ultrasound, abdominal radiography, cystoscopy, cystography, excretory urography and uroexcretory computed tomography can be performed for diagnosis (Fossum, 2021).

Surgical treatment is indicated to ectopic ureter correction. The choice of the surgical technique depends on the type of ureteral ectopia present, of renal function, ureteral insertion site and the coexisting anatomical anomalies (Kosornsri et al., 2020). The definitive resolution for the recurrent bacterial infection process in affected patients is only obtained with surgical correction, since the persistence of the anatomical anomaly favors new infections after the antibiotic therapy ends. The non-correction of the ectopic ureter can trigger renal degeneration due to the pelvis dilation, pyelonephritis and recurrent cystitis (Tambella et al., 2021).

This study aimed to report the diagnostic and clinical surgical conduction of intramural unilateral ectopic ureter correction using neoureterostomy technique in a female dog, 7-months-old, attended at Veterinary Hospital of Federal Rural University of Rio de Janeiro with complaint of urinary incontinence since the 2-months of age and a clinical history of bacterial cystitis.

## Case report

**Figure 1:**
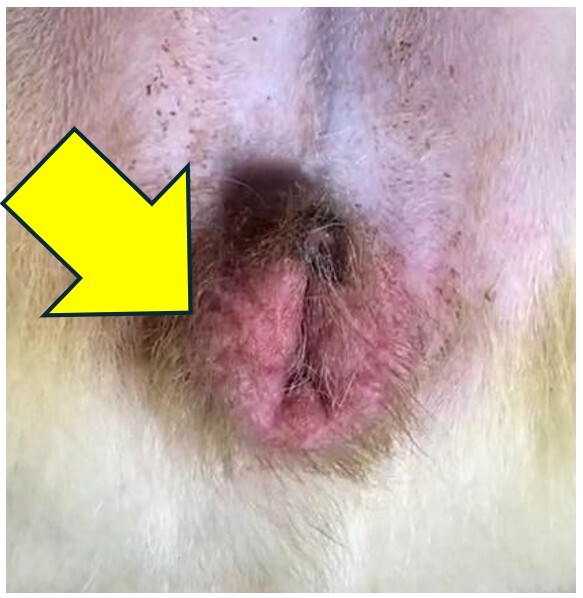
Vulva (ventral view) of dog, female, intact, Siberian Husky, weighing 16 kg, 7-months-old, presenting blushing caused by inflammation secondary to urinary incontinence, attended at the Veterinary Hospital of Universidade Federal do Rio de Janeiro. Font: Image Service at the Veterinary Hospital of Universidade Federal do Rio de Janeiro.

A 7-month-old Siberian Husky, female, intact, weighing 16kg, was attended at Veterinary Hospital of Federal Rural University of Rio de Janeiro with complaint of urinary incontinence since her adoption with 2-months of age and a clinical history of bacterial cystitis. Abdominal ultrasound, performed before the consultation, showed the pelvic urethra measuring 0.25 cm with suggestive diagnosis of ureterocele due to the intravesical dilation of the submucosal portion of the pelvic part of the right ureter. To the physical exam patient presented all the parameters inside normality. In the visual inspection was observed perivulvar dermatitis (Figure 1).

Blood count and blood biochemistry were inside normality. In urinalysis was identified alkaline pH (pH8, ref: 5,5-7,5), increase in red blood cells count, (10 per field, ref: 0-4) and presence of bacteria. In a bacterial culture made with a urine sample obtained by cystocentesis was isolated the microorganism *Proteus mirabillis*, resistant to Cefotaxime, Ceftriaxone, Meropenem, Ceftazidime, Cefepime, Aztreonam; and sensible to Levofloxacin, Amoxicillin with Clavulanic Acid, Enrofloxacin, Ampicillin, Sulfamethoxazole with Trimethoprim. Due to the urinary infection, therapy with Enrofloxacin antibiotic was established (5mg/kg; BID; for 14 days).

**Figure 2:**
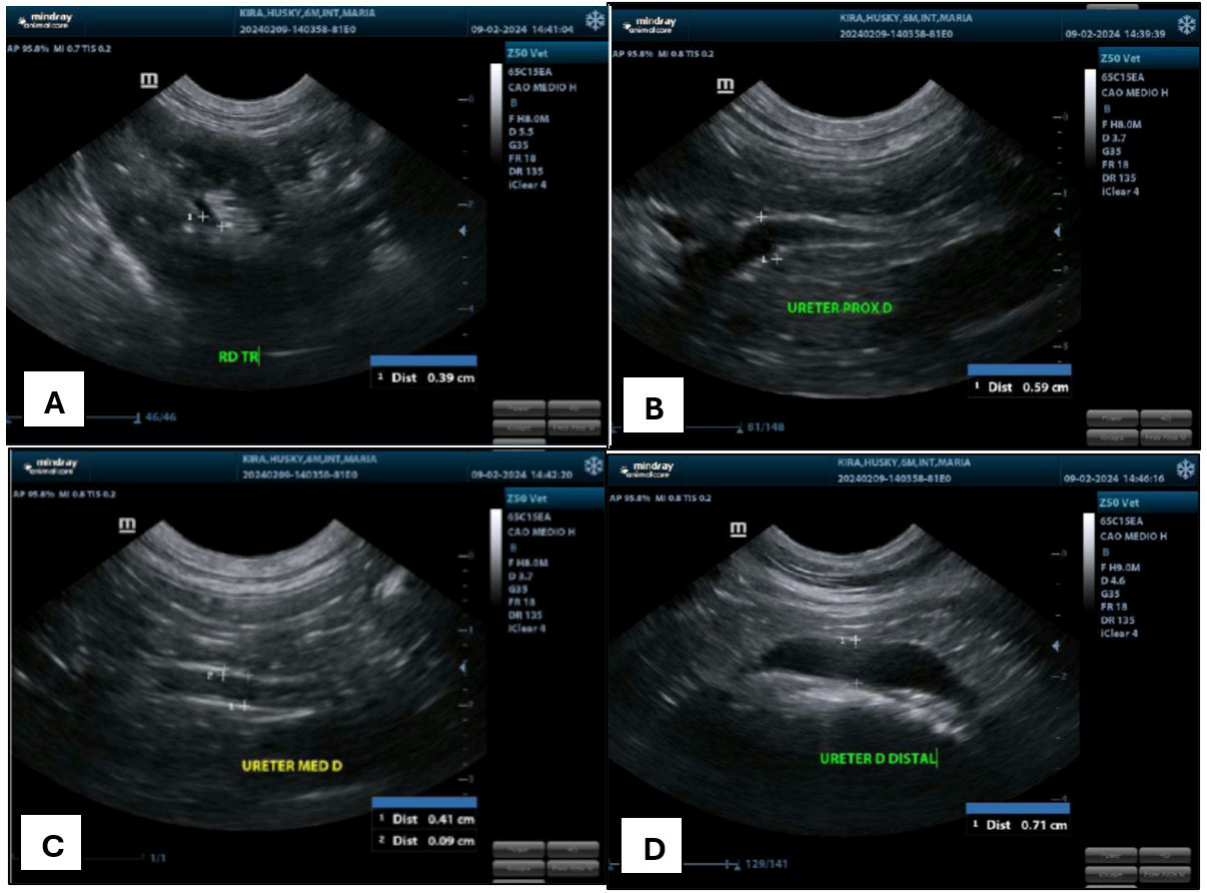
Abdominal ultrasound of urinary tract in dog, female, intact, Siberian Husky, weighing 16 kg, 7-months-old, attended at the Veterinary Hospital of Universidade Federal do Rio de Janeiro. A) Right renal pelvis dilated by anechoic content (0.39 cm); B) Right ureter diffusely dilated with proximal portion measuring 0.59 cm; C) Right ureter medium portion measuring 0.41 cm and thickened ureteral wall measuring 0.09 cm; D) Right ureter distal portion measuring 0.71 cm. Font: Image Service at the Veterinary Hospital of Universidade Federal do Rio de Janeiro.

Urotomography exam was requested but for financial restriction the same could not be performed. That way a new abdominal ultrasound was requested with emphasis in the urinary tract and an excretory urography exam. In the ultrasonography exam (Figure 2), was noted the presence of a tubular structure filled with anechoic content compatible with dilated right ureter entering in the topography of the vesical trigone and traveling a intramural path until the proximal urethra topography. Right renal pelvis dilated by anechoic content (0.39 cm) (Figure 2A). Right ureter diffusely dilated by anechoic content, with its cranial abdominal part measuring 0.59 cm (Figure 2B), caudal abdominal part 0.41 cm (Figure 2C), and the pelvic segment 0.71 cm diameter (Figure 2D). Thickened ureteral wall, measuring 0.09 cm (Figure 2). The ultrasonography diagnosis suspicion was intramural ectopic right ureter.

**Figure 3:**
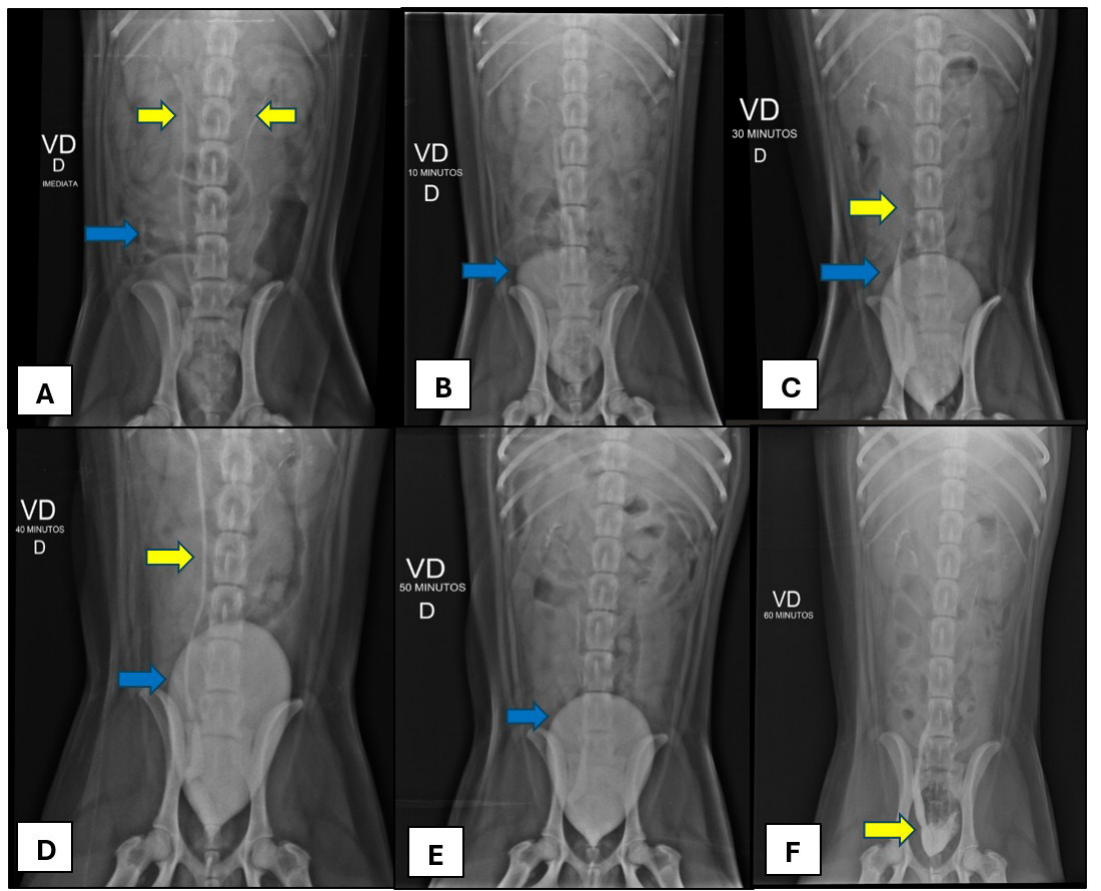
Ventrodorsal projections of an excretory urography exam in a dog, female, intact, Siberian Husky, weighing 16 kg, 7-months-old, attended at the Veterinary Hospital of Universidade Federal do Rio de Janeiro. A) Immediately after contrast administration; B) 10 minutes after contrast administration; C) 30 minutes after contrast administration; D) 40 minutes after contrast administration; E) 50 minutes after contrast administration; F) 60 minutes after contrast administration. A) Presence of radiopaque contrast defining the ureters (yellow arrows) and urinary bladder that is still barely evident (blue arrow); B and E) Radiopaque contrast showing full urinary bladder (blue arrows). C and D) Radiopaque contrast showing full urinary bladder (blue arrows) and right ureter (yellow arrows); F) Empty urinary bladder and radiopaque contrast showing dilated right pelvic ureter (yellow arrow). Font: Image Service at the Veterinary Hospital of Universidade Federal do Rio de Janeiro

Excretory urography was performed using iodinated contrast dose of 600 mg/kg intravenously, highlighting dilated right ureter with atypical anatomical path, caudally to the vesical trigone, suggesting opening close to the urethra (Figure 3).

Given the suspected diagnosis of ectopic right ureter, suggestively intramural, it was opted for the surgical approach. Methadone dose of 2 mg/kg and dexmedetomidine dose of 2 mcg/kg was administered as preanesthetic medication, intramuscular, and propofol induction dose of 1 mg/kg/min (total 2 mg/kg). Periglottic block intubation with lidocaine 2% total volume 1 mL, using endotracheal tube Murphy nº 8.5, maintenance with propofol between 200 and 250 mcg/kg/min, trans surgical analgesia with ketamine 1 mg/kg/h and dexmedetomidine 1 mcg/kg/h. Locoregional lumbosacral epidural block was made with bupivacaine 0.5% dose of 0.26 mL/kg and morphine dose of 0.1 mg/kg. Antibiotic prophylaxis was also performed using cefazolin dose of 25 mg/kg intravenously.

**Figure 4:**
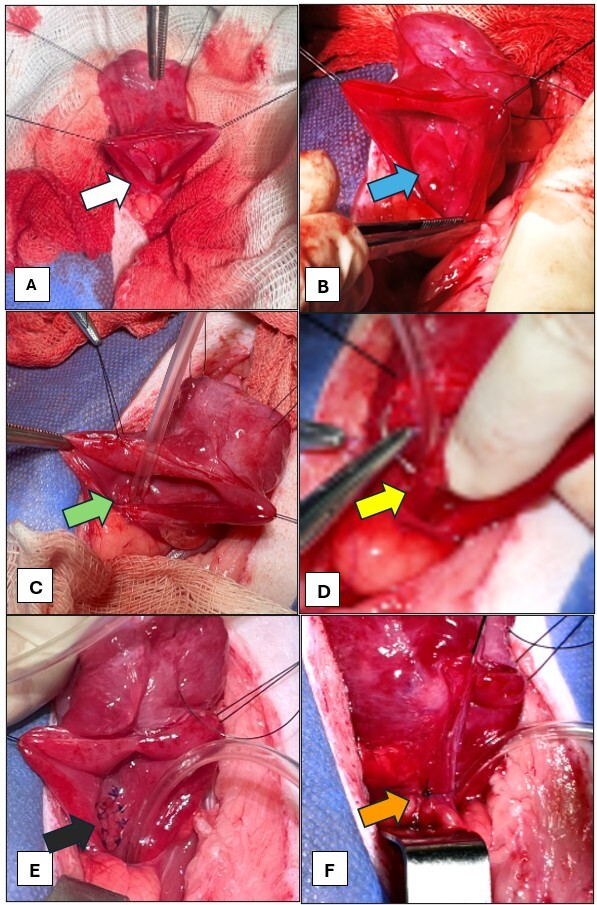
Neoureterostomy surgery to intramural ectopic ureter correction in dog, female, intact, Siberian Husky, weighing 16 kg, 7-months-old, attended at the Veterinary Hospital of Universidade Federal do Rio de Janeiro. A) Opening of urinary bladder (white arrow) ; B) Dilated pelvic ureter (blue arrow); C) Urethral catheterization with probe nº12 (green arrow); D) Suturing all around the newly formed ostium with polydioxanone monofilament absorbable sutures nº 4.0 in simple interrupted pattern (yellow arrow); E) Finished simple interrupted pattern sutures evidencing newly formed ostium (black arrow); F) Right ureter (pelvic part) ligature that opened into the urethra (orange arrow). Font: Image Service at the Veterinary Hospital of Universidade Federal do Rio de Janeiro.

The surgical correction was made by neoureterostomy (Figure 4). Pre-pubic incision, identification and gentle traction of the urinary bladder were made (Figure 4A). It was observed that the right ureter penetrated the vesical wall in the correct anatomic position, however it followed an abnormal intramural path, confirming the diagnosis of ectopic intramural ureter (Figure 4B). Moreover, after ventrocaudal surgical opening of the bladder, it was possible to visualize a submucosal layer dilation of the ectopic ureter compatible with ureterocele. Through identification of the ureterocele intramural portion, its surgical opening was performed with the help of scalpel blade nº 15 and urethral catheterization with probe nº12 (Figure 4C). Then sutures were made all around the newly formed ostium with polydioxanone monofilament absorbable sutures nº 4.0 in simple interrupted pattern (Figure 4D and 4E). Ligature of the pelvic right ureter to the newly formed ostium was also performed using polydioxanone monofilament absorbable sutures nº 4.0 (Figure 4F). Cystorrhaphy was executed on two planes, first in continuous simple pattern and then in Cushing pattern with polydioxanone monofilament absorbable sutures nº 2.0 and posterior omentalization. Celiorrhaphy with polydioxanone monofilament absorbable sutures nº 2.0 in continuous simple pattern was performed, subcutaneous was reduced in mattress pattern with the same thread. Dermorrhaphy was performed in Sultan pattern with non-absorbable nylon sutures 3.0. Concomitantly, ovariohysterectomy was performed to avoid the possibility of hereditary transmission of any anomaly, since the ectopic ureter has congenital origin. 

Post-surgical medication prescribed were Meloxicam (0.05 mg/kg; SID; for 3 days), Dipyrone (25 mg/kg; QID; for 6 days), Tramadol (5 mg/kg; QID; for 7 days), and continued use of Enrofloxacin (5 mg/kg; SID; for 5 days more). 

**Figure 5:**
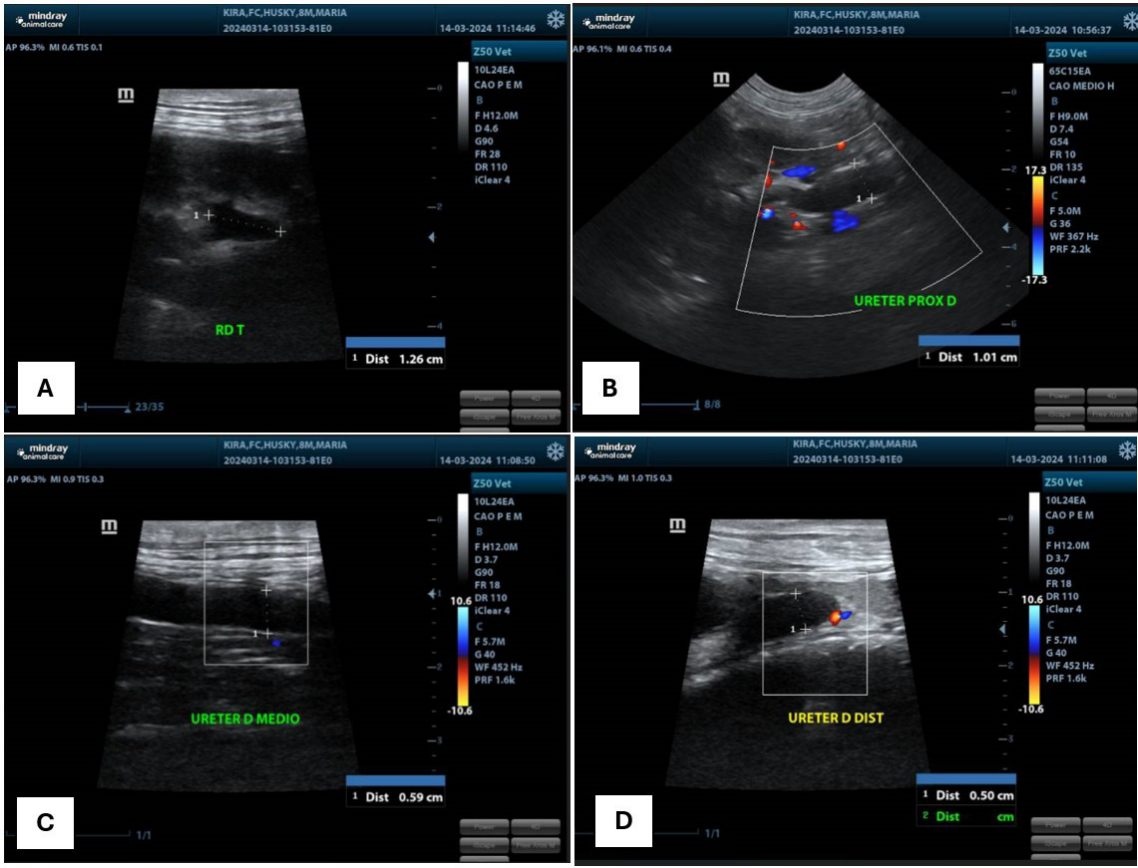
Abdominal ultrasound of urinary tract 2 days after neoureterostomy surgery in dog, female, intact, Siberian Husky, weighing 16 kg, 7-months-old, attended at the Veterinary Hospital of Universidade Federal do Rio de Janeiro. A) Right renal pelvis dilated by anechoic content (1.26 cm); B) Right ureter diffusely dilated by anechoic content, with its cranial abdominal portion measuring 1.01 cm; C) Right ureter (caudal abdominal part) measuring 0.59 cm; D) Right ureter (pelvic part) measuring 0.50 cm. Font: Image Service at the Veterinary Hospital of Universidade Federal do Rio de Janeiro.

Two days after surgery, the patient returned to follow up ultrasound. The animal was alert and active. Ultrasonography exam showed maintenance of ureterocele and right ureteral dilation with renal pelvis even more dilated (0.39 cm to 1.26 cm) (Figure 5A, 5B, 5C and 5D). 

**Figura 6:**
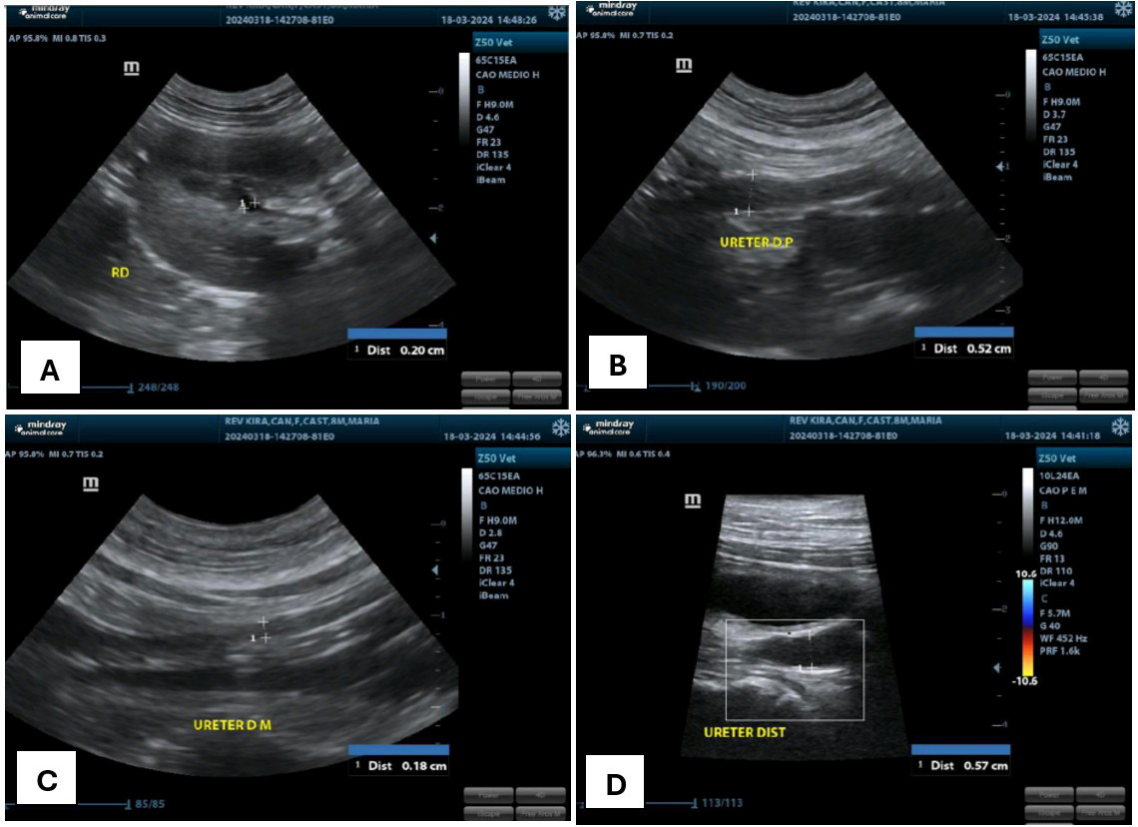
Abdominal ultrasound of urinary tract 6 days after neoureterostomy surgery in dog, female, intact, Siberian Husky, weighing 16 kg, 7-months-old, attended at the Veterinary Hospital of Universidade Federal do Rio de Janeiro. A) Right renal pelvis dilation inside the upper limit, measuring about 0.2 cm; B) Right ureter dilated by homogeneous anechoic content in abdominal and pelvic parts, narrowing on middle third, measuring about 0.52 cm on cranial third; C) Right ureter measuring about 0.18 cm on middle third; D) Right ureter measuring about 0.57 cm on caudal third. Font: Image Service at the Veterinary Hospital of Universidade Federal do Rio de Janeiro.

Six days after surgery a new ultrasonography exam was executed (Figure 6) without any relevant alterations, right renal pelvis dilation inside the upper limit, measuring about 0.2 cm (Figure 6A), right ureter with less evident dilation than observed before surgery (Figure 6C and 6D), showing general improvement. 

Sixty days after surgery, tutor reported that the patient presented significant improvement of incontinence, presenting rare involuntary urination and only when urine was retained for prolonged time.

## Discussion

For being classified as a congenital condition like ureterocele, ectopic ureter diagnosis is normally found when patients are still puppies. Still, according to literature data, young females correspond from 89 to 95% of the reported cases (Acierno & Labato, 2019), as well as observed in the reported case. Related to the breed, Siberian Husky is described by literature as one with the higher genetic predisposition to ectopic ureter together with English Bulldog, Golden Retriever, Miniature Poodles, Labrador Retriever, West Highland White Terrier, Boxer and Newfoundland (McLoughlin & Chew, 2000).

Urinary incontinence associated with bacterial urinary tract infection, as well as perivulvar dermatitis are observed clinical signs in animals with ectopic ureter (McLoughlin & Chew, 2000), as shown in this report, in which the patient presented these symptoms since her adoption with 2-months-old, being diagnosed however only with 7-months-old. It is a belief that later diagnosis happens due to the subtle clinical signs that do not attract tutor attention, especially in unilateral ureteral ectopia cases or with the ureter insertion near to the vesical trigone, which allows the retrograde filling of the bladder leading to mild signs of urinary incontinence or absence of symptoms at all (Tambella et al., 2021).

In 90% of the cases, ureteral ectopia is correlated to several urinary tract anomalies (Bianchi et al., 2013), such as observed in the reported patient, that in addition to the ectopic ureter also presented ureterocele. Animals with urinary tract anomalies are more predisposed to clinical signs of urinary incontinence and recurrent urinary tract infections, since the anatomical deformities predispose the pathogens ascension, being essential the culture exam and antibiogram exam of the patient urine (Weese et al., 2019). A study carried out by Lima et al. (2024) evaluated 233 urine cultures and antibiograms of cats and dogs, noting 8 different genera of microorganisms, being the *Proteus* spp. one of the most predominant in dogs, which was isolated in the patient's urine of the present study, demonstrating sensibility to enrofloxacin in 61.11% of the cases, reason why it was opted for its administration in the reported case.

Patient did not present significant alterations in the blood count and blood biochemistry, as expected in unilateral ectopic ureter patients, once the kidney and the contralateral ureters without alterations compensate the renal function (McLoughlin & Chew, 2000; Tambella et al., 2021). Abdominal ultrasound, in the present report, has satisfactory results as screening method, indicating the presence of ipsilateral hydroureter, ureteritis and pyelectasis. The diagnostic assistance of excretory urography and abdominal ultrasound were important in the ureteral ectopia identification, however definitive diagnosis and the differentiation between intra or extramural ureter only happens during the intraoperative. The surgical exploration is fundamental to determine the exact type of ectopic ureter present when the preoperative diagnosis is inconclusive (Fossum, 2021). Cystoscopy and computed tomography exams would be the chosen methods to Samii et al. (2004) and Hoelzler & Lidbetter (2004), for having a greater degree of visualization of the anatomy involved, but because of financial restrictions it was not possible to be executed in the reported case.

Surgical correction is the golden pattern treatment, and the choice of the technique will depend on the type of ectopic ureter, intra or extramural (Kosornsri et al., 2020). Neoureterostomy procedure was chosen for being less traumatic, have shorter surgical time and no need for excision and reimplantation of the ureter when compared to ureteroneocystostomy technique. An even less invasive approach will be the ureterostomy guided by cystoscopy, but this requires specific equipment (Fossum, 2021).

The surgical technique was considered simple, fast and easy to execute, being performed as described by Fossum (2021). For occlusion of the distal ureter to the surgical ostium, is recommended using one or two sutures with non-absorbable thread, although in the present report it was opted for the use of polydioxanone-PDS (absorbable monofilament of long-absorption) due to the contact risk of the thread with the lumen of the urinary tract, being the PDS a safe thread, non-calculogenic (as the Nylon, for example) and low capillarity.

There was an important volume increase of the right renal pelvis observed in abdominal ultrasound two days after surgery, suspect of possible ureteral obstruction. This is a common and even expected complication because the urethra swells after surgical manipulation, which can cause temporary obstruction of the urethral flow (Fossum, 2021). A reported complication is postoperative stenosis at the ureteral implantation site, resulting in urinary reflux, and new intervention may be indicated (Reichler et al., 2012). With favorable evolution after six days post-surgery, the possibility of a permanent post-surgical complication was discarded.

The most common neoureterostomy complications include hydronephrosis, cystitis, hydroureter, dysuria, and the persistent urinary incontinence as the most often complication after the surgical procedure, occurring approximately in 44 to 66% of the animals (Tambella et al., 2021). According to Russell et al. (2023), presence of lower urinary tract infection is significantly associated to urinary incontinence in the immediate and late postoperative. To Ho et al. (2011), both lower urinary tract infection and hydroureter were not relevant to the development of urinary incontinence in immediate and late postoperative. Urinary incontinence can be treated clinically with the use of phenylpropanolamine, combined in some doses with canifedrine, flavoxate, estriol, or endoscopic injection of collagen deposits into the submucosa of the proximal region of the urethra, but its effects are controversial (Reichler, et al., 2012).

In the present report, despite the presence of hydroureter and lower urinary tract infection previously to the surgery, there was a gradual and significant improvement of the urinary incontinence over 60 days after the surgical procedure, and tutor only reported rare episodes of involuntary urination, above all when the patient retains urine for prolonged periods.

## Conclusion

The early and precise diagnosis, using the abdominal ultrasound and excretory urography tools, and the adequate surgical technique were important factors to the resolution of the urinary incontinence, despite the previously presence of hydroureter and ureterocele, and right kidney patient preservation. Right renal pelvis dilation was an expected complication, however concerning in the immediate postoperative, and the abdominal ultrasound was a simple and fundamental tool for its monitoring.
